# Dealing with Alcohol-related problems in the Night-Time Economy: A Study Protocol for Mapping trends in harm and stakeholder views surrounding local community level interventions

**DOI:** 10.1186/1756-0500-4-204

**Published:** 2011-06-18

**Authors:** Peter Miller, Darren Palmer, Nicolas Droste, Jenny Tindall, Karen Gillham, Anders Sonderlund, Emma McFarlane, Florentine de Groot, Amy Sawyer, Daniel Groombridge, Christophe Lecathelinais, John Wiggers

**Affiliations:** 1 School of Psychology, Deakin University, Geelong, Victoria, Australia; 2 School of History, Heritage and Society, Deakin University, Victoria, Australia; 3 Hunter New England Population Health, Newcastle, Australia; 4 School of Medicine and Public Health, The University of Newcastle, Newcastle, Australia

## Abstract

**Background:**

This project will provide a comprehensive investigation into the prevalence of alcohol-related harms and community attitudes in the context of community-based interventions being implemented to reduce harm in two regional centres of Australia. While considerable experimentation and innovation to address these harms has occurred in both Geelong and Newcastle, only limited ad-hoc documentation and analysis has been conducted on changes in the prevalence of harm as a consequence, leaving a considerable gap in terms of a systematic, evidence-based analysis of changes in harm over time and the need for further intervention. Similarly, little evidence has been reported regarding the views of key stakeholder groups, industry, government agencies, patrons or community regarding the need for, and the acceptability of, interventions to reduce harms. This project will aim to provide evidence regarding the impact and acceptability of local initiatives aimed at reducing alcohol-related harms.

**Methods/Design:**

This study will gather existing police data (assault, property damage and drink driving offences), Emergency Department presentations and Ambulance attendance data. Further, the research team will conduct interviews with licensed venue patrons and collect observational data of licensed venues. Key informant interviews will assess expert knowledge from key industry and government stakeholders, and a community survey will assess community experiences and attitudes towards alcohol-related harm and harm-reduction strategies. Overall, the project will assess: the extent of alcohol-related harm in the context of harm-reduction interventions, and the need for and acceptability of further intervention.

**Discussion:**

These findings will be used to improve evidence-based practice both nationally and internationally.

**Ethical Approval:**

This project has been approved by Deakin University HREC.

## Background

Alcohol-related problems are a major cause of social disorder and illness in Australia. In particular, problems associated with the night-time economies of urban and regional centres cause substantial community concern and constitute a substantial drain on police, community and health resources. The estimated cost of alcohol to the community is $15.3 billion including crime, violence, treatment costs, loss of productivity and premature deaths in 2004/05 [1]. Alcohol has also been identified as a factor in approximately three quarters of assaults and offensive behaviour on the street [2]. Similarly, alcohol at or over 0.05 g/100 ml (%) was found to be present in 29.1% of all drivers in fatal accidents in Australia [3]. High-risk alcohol consumption causes over 400 road deaths and 7,700 serious road injuries requiring hospitalisation each year, at an estimated cost to the community of over $1.34 billion [4].

Previous research has identified a number of issues that contribute to the levels of short-term harm associated with risky drinking. These include: excessive consumption at licensed venues, consumption in public areas and the lack of transport and security in entertainment precincts [5,6]. Over half of all offences occurring on the street have been associated with licensed venues in Australia [2]. Factors which increase risky drinking and associated harms on licensed venues are complex and include: aspects of patron mix; levels of comfort, boredom, alcohol service, and intoxication; environmental factors such as noise, light and seating; promotions that cause mass intoxication; and the behaviors of bouncers [5]. Violence has also been shown to be perpetuated by poor management, lax surveillance and enforcement by liquor licensing, lack of transport options for patrons, and inappropriate bureaucratic controls and legislation [5].

The problems associated with alcohol consumption and licensed venues are well documented. However, there is a dearth of research that identifies which interventions work in which settings and on what aspects of the problems. This study will seek to gain an in-depth understanding of changes in levels of harm in the context of interventions being delivered and how they are viewed by different stakeholders. By looking at different cities, we will also be able to ascertain how different cultural elements might influence the effectiveness of different interventions. This paper describes the methodology to be used in this study.

## Methods/design

### Study Aim

Managing the consumption of alcohol and its associated harms within and around licensed venues is a challenge for police and communities nationally and internationally. This study aims to provide a comprehensive investigation into some innovative policing and community-based interventions focused on licensed venues in Geelong and Newcastle.

### Study Design

The study will incorporate a number of study designs including cross-sectional, longitudinal and observational. The study will incorporate a blend of epidemiological and social research methods:

1. Patron intercept surveys

2. A computer assisted telephone community survey (CATI)

3. Key informant interviews

4. Covert observations of licensed venues

5. Secondary data analysis (accident and emergency attendances, assault and property damage offences and ambulance attendances).

The trial is funded by the National Drug and Law Enforcement Fund. Ethical approval to conduct the study has been obtained from the Deakin University Human Research Ethics Committee (EC 41-2009).

### Setting

The study will be undertaken in two regional Australian cities located in separate states (Geelong, Victoria and Newcastle, New South Wales). Both cities are regional centres located within 150 km of their state capital city. The populations of the cities are 211,841 (Geelong) and 288,732. Both cities have a higher median age, a lower median individual income and higher unemployment compared to the Australian population[7].

### Samples

#### 1. Patron intercept surveys

In both cities, the sample will comprise of patrons attending licensed venues (hotels and nightclubs) located within the main entertainment precincts of the regional cities. Approximately 4000 patrons (2000 per city) will be randomly selected (convenience sample) over 36 nights during the study period.

#### 2. Community survey

In both cities, the study area will be defined as the Local Government Area (LGA) in which the main entertainment precinct was located, plus adjoining LGAs. In each city, a sample of 1250 telephone numbers and their corresponding names and addresses will be randomly selected from telephone directories. Mobile and business numbers will be excluded from the samples. Household members with the next birthday in the selected households who are aged 18 years or over, are able to converse in English and reside in the study area will be eligible to participate in the survey.

#### 3. Key informant interviews

The study will aim to conduct 100 initial in-depth interviews with identified local key informants including police, licensees, taxi drivers, health, ambulance, security personnel, licensing authorities, councils, general practitioners, pharmacists and alcohol and other drug workers (50 for each site). Up to 50 follow-up (25 each site) in-depth key informant interviews will be undertaken at a later stage in the study.

#### 4. Venue observations

In both cities, the study area will be defined as licensed venues (hotels and nightclubs) located within the main entertainment precincts of the regional cities. The sample will comprise of 12 venues located in Geelong and 12 venues located in Newcastle. Each venue will be observed on at least six occasions over a 15 month period (approximately every 3 months).

#### 5. Secondary data analysis

These data sources will include:

• Victorian Police and NSW Police data on arrests, incidents, drink driving offences (Vic only) and Alcohol and Other Drug-related domestic violence;

• Emergency Department presentations from Geelong Hospital and two local hospitals in Newcastle (John Hunter Hospital and the Calvary Mater Hospital);

• Ambulance attendances on alcohol and other drug/violence related calls - Geelong only (Limited ambulance attendance data is available in NSW).

In addition to the collection of this existing data, the research team will work with relevant agencies to gain the highest quality data within practical limits as well as developing links for data transfer and storage.

### Data collection Procedures

#### 1. Patron intercept surveys

Patron surveys will be conducted longitudinally over an 18 month data collection period at randomly selected venues on a fortnightly basis (36 nights). In agreement with licensees, a team of 4 or more researchers will attend up to 6 venues on allocated evenings. Each team will be allocated a 'Team Leader' who will be responsible for liaising with the venue staff upon entry, identifying interview locations, managing the behaviour and performance of the interviewers, and monitoring safety.

Each member of the research team will randomly approach patrons, briefly explain the survey and invite them to participate in a 5 minute survey. Both consent and non-consent will be recorded on the PDA. Each respondent will be given a unique identifier based on their personal details (such as the first 3 letters of their first name, month/year of birth/last 3 letters of their occupation).

At some point during the survey, a business-sized information card will be provided to each respondent. The business cards will have a study internet address and contact details if respondents wish to know more about the study or withdraw their consent.

All surveys will be completed on busy nights of the week (typically Friday and Saturday nights) between peak hours (typically 9 pm to 1 am). Surveys will be conducted at later times (until 5 am) in Geelong on at least 2 occasions to reflect the later trading hours. Each fortnight, the research team will survey up to 100 patrons inside/external to consenting venues located in the cities. Survey data will be directly entered into Personal Digital Assistants (PDAs) or Palm Pilots. The data will then be extracted for data analysis.

#### 2. Community survey

An invitation letter will be mailed to all randomly selected households. The households will be contacted by telephone and the eligible household member will be asked to complete a computer-assisted telephone interview at that or a later time. The interview will consist of 165 items (approx 25 mins) and will be developed based on questions from the British Crime Survey, the ABS Personal Safety Survey and the AIHW National Drug Strategy Survey. The interview will be pilot tested with respondents from a sub-sample of the selected study sample with subsequent changes being made to the survey.

Experienced telephone interviewers will contact each household between 9 am and 8 pm Monday to Friday, and between 10 am and 4 pm on Saturdays (if necessary). A maximum of ten contact attempts will be made for each selected number/household member.

#### 3. Key informant interviews

A research assistant will contact each respondent in the sample, provide a brief description of the study and interviews, and invite them to participate. Following consent, an appropriate interview time will be scheduled. Interviews will be tape-recorded, unless the participant objects, and all interviews will be transcribed and returned to the interviewee for review. Interviewees will be able to correct any errors in transcribing and also remove any sections they do not wish reported. In addition, they will be able to add or expand on any points. In following an in-depth qualitative methodology, key informants will be asked questions based on a series of prompts, rather than a strict set of questions [8,9]. This is designed to allow the researcher and the participant to follow any points that may arise and to modify questions accordingly. Key issues and themes will be identified through discussions with these key informants.

#### 4. Venues observations

A researcher from the study will contact all venues in the sample and arrange an appropriate meeting time. During this meeting, the researcher will provide a brief description of the study and the observations, provide a copy of the observation survey, and invite them to participate. Written consent will be sought from all venues.

All late night venues located in both cities will be covertly observed during busy nights of the week (typically Friday and Saturday nights) during peak trading hours (between typically 10 pm and 5 am). The venues will not be informed when the observations will be conducted. Teams of at least two observers will independently observe each venue to allow for inter-rater reliability testing of the observation survey and the measures. In addition, independent quality assurance observations will be conducted on at least 10% of observations. These observations will be conducted by experienced staff at the same time as the venues observations, and the data will then be analysed for inter-rater reliability against the observational data.

All observation and quality assurance staff will be trained in the Responsible Service of Alcohol by an accredited trainer, and observation procedures. Data will be entered into a Personal Data Assistant (PDA) or Palm Pilot. The data will then be extracted for data analysis.

#### 5. Secondary data analysis

Data collection will follow different protocols for each of the different sources listed below. Variability in data systems and measures will exist due to variations between Australian states.

##### 1. Emergency Department attendances

Emergency department level data will be downloaded from the Barwon Health Geelong Hospital (Geelong), John Hunter Hospital (Newcastle) and the Calvary Mater Hospital (Newcastle). Where possible, identical de-identified data will be accessed and analysed for Emergency Department (ED) presentations across the sites. Following the methods outlined by Young and colleagues [10] in their work from the International Collaborative Study of Alcohol and Injury (the Emergency Room Collaborative Alcohol Analysis Project or ERCAAP), cases selected will be based on International Classification of Diseases (ICD) codes for all injuries (S00-T98 or ICD9 800-999) for two late-night periods which were identified as having particularly high levels of alcohol involvement: a nine-hour period between 10:00 pm and 6:59 am (Night 1) and a five-hour period between 12:00 midnight and 4:59 am (Night 2) [10]. Young et al. reported that such injury presentations constitute 21.2% and 9.7% respectively of all presentations, and include 46% and 56% respectively of cases with prior alcohol involvement. The former (Night1) has the advantage of a higher volume of cases and hence more statistical power in most applications while the latter has the advantage of greater specificity and hence less sensitivity to external bias [10].

##### 2. Police Offences

Offence data will be downloaded from NSW and Victoria Police databases at a unit record level. All data will be de-identified. Offences covered will include: Assaults, Property Damage (Geelong only), Street Offences (Newcastle only) and Drink Driving (Geelong only). Data fidelity will be assured by communication between sites to ensure that data being compared is comparable. For example, although Victoria and NSW police classify assaults differently, the research team will ensure that specific types of assault are matched (e.g. Grievous Bodily Harm) and others not related to the study are excluded (e.g. Obstruct Crew of Ship -Execution of Duty, which is an assault offence in Victoria).

##### 3. Ambulance Victoria attendance

Ambulance Victoria data will be collected from 1 August 2008 for the Geelong and Surf Coast region. No electronic records were available prior to this date. De-identified data will be accessed for all alcohol and other drug (AOD)-related cases attended by ambulance paramedics in the Geelong and Surf Coast region from 1 August 2008 - 31 July 2011. General demographic data relating directly to the research aims of identifying the nature of people experiencing harm in the night-time economy and the situational factors involved will be collected. No reference will be made to specific addresses or venues. Two forms of data will be accessed: Call out data (CAD; Computer Assisted Dispatch) and Individual treatment records for patients (Victorian Ambulance Clinical Information System; VACIS). Any case which includes text description of any of the following will be analysed: assault, etoh, alcohol, cannabis, marijuana, ecstasy, amphetamine, speed, methamphetamines, ice, heroin, ketamine, GHB, PBT, blood alcohol, overdose and OD. Cases will be classified by at least two research associates and inter-rater reliability will be assessed.

### Measures

#### 1. Patron intercept surveys

The Patron survey will consist of 7 domains:

1. Demographics

2. Limited demographic details will be obtained, including first name, year of birth, postcode of residence and occupation. These will allow the identification of repeat interviewees, without being identifiable.

3. Past and planned movements on the survey night

4. Details on the interviewee movements throughout the night (i.e. places visited), amount of money spent, motivation for going out, how they are planning to get home and how convenient this is for them.

5. Normal entertainment patterns

6. Interviewees will also be asked about how often they normally go out to licensed venues, how often they become intoxicated and how often they are refused service in licensed venues.

7. Safety

8. Interviewees will be asked about their perceptions of safety in the venue they are attending and what measures they use to keep safe. They will also be asked about how often they have seen police and whether their ID has been checked.

9. Experience of harm

10. Interviewees will be asked about their experience of harm, particularly whether or not they have witnessed or been involved in aggressive or violent incidents in the past 12 months. They will also be asked details about any events they report, such as levels of intoxication and incident setting.

11. Policy attitudes

12. Interviewees will be asked for their attitudes towards a number of policy measures currently in place in Geelong and Newcastle, and be asked to gauge their effectiveness.

13. Patron intoxication

14. Finally, interviewees will be asked to rate their level of intoxication. They will also be asked about how much they have had to drink during the night, how much they drank before attending a licensed venue and whether they have been refused service that night. Interviewees will also be asked about any other substance use. Patron intoxication will also be independently observed and rated by the interviewer at the end of the interview.

Details will also be recorded about the location of the interview, time, date and the interviewer's name.

#### 2. Community survey

The survey will include four domains:

##### 1. Perceptions and experiences of crime and safety

Respondents will be asked a range of questions pertaining to their perceptions of safety and experiences of crime in the entertainment precinct of their city. They will be asked:

• The degree to which they believed alcohol is a problem, is a major contributor to crime and consumption at venues contributes to a large percentage of the crime in the precinct (6 point Likert Scale - strongly agree to strongly disagree).

• To estimate the percentage of crime in the precinct they think is alcohol-related (%).

• To indicate if they believed that there are problems with crime or people creating a public nuisance (yes, no, don't know, refused).

• To indicate which of 11 types of crime are a problem in the precinct (burglaries, car theft, other theft, Louts/gangs, prowlers/loiterers, drunkenness, vandalism/graffiti, dangerous driving, illegal drugs, sexual assault, other assault).

• To indicate which of 7 types of problems commonly related to drunk or rowdy people are occurring in the precinct (verbal abuse, physical abuse, fighting between intoxicated people, noise/disturbances, intoxicated people begging, alcohol-related vandalism, homeless or alcoholics drinking on the streets). They will be asked which one is the most frequently occurring, and how often they have seen or experienced it in the precinct in the last year (every week, once/twice per month, every few months, less often, not in last year, never).

• To indicate their feeling of safety when walking or waiting for public transport alone after dark in the precinct (very unsafe, unsafe, neither safe nor unsafe, safe, very safe, never do). If they felt unsafe, they will be asked for the main reasons.

##### 2. Awareness and attitudes towards local strategies in city entertainment precincts

For each strategy, the following will be measured:

• Awareness of strategy (yes, no, don't know, refused)

• If aware, level of support for the strategy (6 Point Likert Scale- strongly support to don't know enough to say).

• If supportive, the main reasons for support

• If not supportive, the main reasons for opposition

• Perceived effectiveness of the strategy in reducing alcohol-related crime in the precinct (4 Point Likert Scale very effective to not effective)

Respondents will be asked to indicate if they have visited a licensed venue in the main entertainment precinct after 10 pm at night in the last year (yes, no, don't know, refused). Respondents who indicate that they have will be asked to indicate:

• How effective the strategies have been on making streets safer and making venues safer (6 point Likert Scale - very effective to not effective).

• Which strategy has had the greatest impact on alcohol-related crime (7 strategies for both areas).

• Whether they think alcohol-related crime has change in the last year (yes, no, don't know), and how they think it has changed (a lot more, a little more, a little less, a lot less, more frequent, less frequent, more aggressive, less aggressive)(Multiple choice).

• Whether the number (more people, less people, no) or the demographics (no change, more males, more females, more older people, more younger people) have changed in the last year (Multiple choice).

• Whether the lock-out has been effective in reducing the number of people on the street (5 point Likert Scale- very effective to not out that late)(Newcastle only).

• Whether early closing has been effective in reducing the number of people on the street (5 point Likert Scale- very effective to not out that late)(Newcastle only).

• Whether there is adequate transport at closing time (yes, no, not out late late).

##### 3. Support for alcohol harm reduction strategies

All respondents will be asked to indicate their level of support for 15 harm reduction strategies using a 6 point Likert Scale (strongly support, support, neutral, oppose and strongly oppose, don't know).

The strategies will include: increasing the price of alcohol, raising the legal minimum age, increased penalties for venues and staff, restricting venues density in high-risk areas, reducing trading hours in high-risk areas, restricting late-night trading in high-risk areas, increasing visible police checks of venues, restrictions on discounted alcohol and promotions, police asking intoxicated offenders where they consumed their last drink, legal liability, ID scanners, Closed Circuit TV, improved communication between venues, mandatory Responsible Service of Alcohol Training (Geelong only), increasing the number of secure taxi ranks, increasing the number of taxis, more severe penalties for drink driving, lowering the legal blood alcohol limit for driving, increasing visible Random Breath Testing, banning alcohol advertising on TV, banning alcohol sponsorship of sporting events, service of low-alcohol drinks at events, mandatory lock-outs and closing late-night venues earlier.

##### 4. Respondent characteristics

The study area (Geelong, Newcastle) in which the respondent is located will be obtained from the sampling frame. Respondents will be asked to provide information regarding their gender, date of birth, Indigenous status, occupation, educational qualifications and income. In addition, respondents will be asked about their frequency of alcohol consumption (never, monthly or less, 2 to 4 times a month, 2 to 3 times a week, 4 to 6 times a week, everyday), the number of standard drinks they typically consume (1-2, 3-4, 5-6, 7-9, 10 or more) and how often they have 6 or more drinks on one occasion (never, less than monthly, monthly, weekly, daily, almost daily).

#### 3. Key informant interviews

As mentioned previously, the key informant interviews will be semi-structured and the interview schedule will be used to guide discussions, rather than necessarily having every question be asked separately. However, the interviewer will have topic prompts for every subject area to ensure all topics of interest are covered.

The Patron survey will consist of 5 domains:

##### 1. Interviewee Details

Key informant details will be recorded, including their name, employer/occupation, how long they have been in the role and how long they have worked in the field. They will also be asked about whether they deal directly with the public, and if so, the type of people they work with and the trends they have observed. They will also be asked about general trends in relevant topics such as intoxication, violence, other social and health harms and any recent changes they have seen in such trends.

##### 2. Current local issues

Key informants will be asked about the local issues they encounter. Specific discussion points will include: underage drinking; intoxication related to licensed venues; responsibility for monitoring intoxication levels at venues; and, the nature and extent of different strategies in their local area which have impacted on their community. They will also be asked about their perceptions of alcohol-related drunkenness, levels of violence, current policing levels and current liquor legislation.

##### 3. Awareness of/Attitudes to current local interventions

Key informants will be asked to discuss study-specific topics related to each of the interventions being implemented in Newcastle and Geelong. The interventions discussed will be:

• Responsible Service of Alcohol strategies (Newcastle)

• Drink restrictions (Newcastle)

• Ceasing service 30 minutes prior to closing time (Newcastle)

• Late-night radio networks (Geelong)

• Secure taxi ranks (Geelong and Newcastle)

• ID scanners (Geelong)

• Closed circuit television (CCTV)(Geelong)

• Accreditation schemes (e.g. 'tick of approval' 'best bar none')(Geelong)

• Replacement of glass containers (Geelong)

• Liquor Licensing Accords (Geelong and Newcastle)

• Lock outs (Geelong and Newcastle)

• Reduced trading hours (Newcastle)

Topics discussed will include: perceptions of effectiveness, advantages and disadvantages of each intervention, and possible avenues for improvement.

##### 4. Illicit drugs

Interviewees will also be asked to discuss what illicit drugs they think are involved in the night-time economy and the different ways in which each drug plays a role. Other topics addressed will include:

• Which illicit drugs are seen most often amongst patrons in the night-time economy?

• Which illicit drugs they think cause the most problems?

• What types of problems are associated with these drugs? (Severity scale of 1-10)

• Which illicit drugs they see mixed with alcohol?

##### 5. Other changes in last 12 months

Key informants will also be asked about other major changes that have impacted on alcohol-related harm in the last 12 months. These can include the economic recession, new venues, closure of venues, changes in Government and changes in police personnel. They can be changes at either the local, state or national level.

##### 6. Crime

Key informants, and police in particular, will be asked about any changes in the type/amount of crime being observed in the last 12 months, including those which are not related to alcohol, but might indicate other crime trends which could impact on alcohol-related crime (e.g. knife crime, gang crime etc).

##### 7. Closing questions

Finally, key informants will be asked a number of quality assurance questions such as:

• Generally, from where do you get the information you have provided us with today?

• How certain are you of your knowledge? (Very certain - moderately certain - a little unsure - very unsure)

In addition to the generic questions, specific questions will also be asked of licensees and police, drawing on their specific expertise and experiences. For example, licensees will be asked about the most popular drinks sold; amounts of drinks people are generally consuming; changes in the speed people are generally drinking, and peak trading hours.

Police will be asked specific questions relating to: operations they have implemented/been involved in the past 2 years; operations are they currently implementing; main features of these operations; how the current operations differ from previous operations; and, what operations they think have been successful/unsuccessful and why.

#### 4. Venues observations

The observation survey will incorporate 10 domains. All results will use observations as the denominator and will be reported as the proportion of all observations. The measures will include:

• Entry procedures: 'proof of age' always checked at the door, observers 'proof of age' checked, door staff refused entry (eg. underage, intoxicated), and all entrances were monitored by staff.

• Patron characteristics: equal mix of gender, and an equal mix of 'under 25yrs' and 'over 25yrs'.

• Patron intoxication levels: no signs of intoxication were observed, and observers witnessed an intoxicated patron attempting to purchase a drink and staff refused service and/or asked the patron to leave.

• Staff characteristics: adequate numbers of bar staff during peak time (based on patron numbers) and observed bar staff being polite/friendly.

• Responsible Service of Alcohol practices: substantial food available when alcohol is served, identified Responsible Service of Alcohol Marshall, serving more than 4 drinks at a time per patron, free water stations on all bars, no stockpiling of drinks by patrons, no drink promotions that encourage excessive consumption, service of drinks in plastic containers, refused to serve a double nip of alcohol, refused to serve a 'Ready-to-Drink' with more than 5% alcohol (Newcastle only) and refused to serve a 'shot' of alcohol (Newcastle only).

• Security measures: adequate ratio of security staff to patrons, electronic ID scanner at the main entrance, and Closed Circuit TV cameras inside and/or outside.

• Safe transport options: designated driver program, staff allowed to call taxis, courtesy transport, advertised secure taxi rank, and advertised the 'Nightrider' bus (Geelong only).

• Crowd control measures: door charges and lock-out/curfew.

• Physical environment: limited crowding around the bar service area, moderate to high level of cleanliness, moderate to high level of lighting, acceptable level of noise, and fair to good flow of traffic.

• Social environment: low levels of sexual activity, no physical or non-physical arguments/fights witnessed, and no signs of illegal drug use.

• Closing procedures: ceasing service at least 30 minutes prior to closing time, actions to inform patrons of closing, and no stockpiling of drinks/allowing patrons to leave with alcoholic drinks/consumption of alcohol in the immediate surrounds of the venues.

#### 5. Secondary data analysis

Measures for secondary data will consist of unit record data relevant to the specific type of information. In addition, Police data will also include Offender and Victim data. This data will be de-identified and accessed in an aggregated form to protect privacy.

The measures will be:

• The number of night-time (Night 1 and Night 2) injury-related emergency department presentations (both sites)

• The number of night-time non-domestic assaults (both sites)

• The number of night-time street offences (Newcastle)

• The number of drink driving and malicious damage offences (Geelong)

### Analysis

The data collected from multiple sources will be triangulated for cross-validation and interpretation purposes. Triangulation is a widely used method of data synthesis which is based on the premise that one can be more confident with a result if different methods lead to the same result [11-13]. If an investigator uses only one method, the temptation is strong to believe in the findings. If an investigator uses two methods, the results may well clash [9]. By using three methods to get at the answer to one question, the hope is that two of the three will produce similar answers, or if three clashing answers are produced, the investigator knows that the question needs to be reframed, methods reconsidered, or both. The method has proved particularly popular in the monitoring of substance use and related trends [e.g. [14-21]]. For the data on experiences, attitudes and practices, simple frequency counts will be tabulated. Raw data from open-ended questions and in-depth interviews will be analysed descriptively via a combined method of open coding and content/theme analysis. Previous work has shown the influence that different types of data can have when interpreting findings [22]. For instance, qualitative data can often point analysis towards certain phenomena in quantitative data or conversely, narratives from qualitative data can often be very useful in describing certain quantitative trends in the words of study participants [23].

All statistical analysis will be conducted using appropriate software (e.g. STATA, SPSS (SPSS v17.0 or later) or SAS/STAT (SAS/STAT System for Windows Release 9.2, March 2008).

#### 1. Patron intercept surveys

The data collected from the surveys will be analysed based on frequency counts, but will also be investigated longitudinally to ascertain any changes in average perceptions of safety or pre-drinking. Group differences (such as different venues, time periods or differences between sites) will be explored using both bi-variate (chi-square) and multivariate statistical methods (logistic regression) to adjust for socio-demographic and geographic differences.

#### 2. Community survey

The data collected in this survey will be analysed by producing frequency counts. Where appropriate, differences will be assessed between the following groups:

• Respondents located in Newcastle and Geelong

• Respondents that have visited a licensed venues located in the main entertainment precinct after 10 pm in the year prior to the survey, and those that have not

• Respondents that reside in the inner-city and outer-city areas

Differences will be assessed for statistical significance using chi square or Fishers Exact Tests.

#### 3. Key informant interviews

Responses from key informants will be analysed primarily based on questionnaire structure and subsequent analysis of narratives using thematic analysis. Thematic analysis (or 'narrative analysis') is an inductive design where, rather than approach a problem with a theory already in place, the researcher identifies and explores themes which arise during analysis of the data [24]. In this analysis, once a theme has become evident, all transcripts will be re-analysed for appearances of the theme. Categorisation will not be exclusive and some narratives may appear in many themes. Categories will be added to reflect as many of the nuances in the data as possible, rather than reducing the data to a few numerical codes [25]. All the data relevant to each category will be identified and examined using a process called constant comparison, in which each item is checked or compared with the rest of the data to establish analytical categories. For the sake of transparency, results reported will be enumerated [26]. Where available, narratives which present opposing viewpoints will also be presented [27].

#### 4. Venue observations

The data collected during these observations will be analysed by producing frequency counts of the specific variables. Data will be analysed based on the number of observations across the study period (approximately 280 observations).

Group differences (such as different venues or time periods) will be explored using both bi-variate (chi-square) and multivariate statistical methods (logistic regression) to adjust for socio-demographic and geographic differences

#### 5. Secondary data analysis

Analysis of secondary data will focus on:

1. Tracking trends over time

2. Comparing differences between cities

Where possible, the analysis of secondary data will be undertaken using time series analysis techniques. In so doing, these techniques will attempt to model the patterns evident in the indicator data and then model any effects of different community interventions evident in a visual inspection of the data. In cases where time series analysis is either impossible or inappropriate (e.g. where fewer than 50 data points are available for a given series), comparisons of 12-month periods over the length of the research period will be undertaken using appropriate statistical techniques (e.g. t-tests for continuous data). Comparative analyses will be conducted using t-tests, anovas, negative binomial regressions and modeling into a generalized estimating equation (gee) framework [28].

## Discussion

This project has the potential to have a substantial impact on law enforcement, government and local community responses to alcohol issues by documenting whole of community measures of harm over time, as well as stakeholder attitudes, both in the context of interventions to reduce alcohol-related problems in licensed venues. In most towns and cities, a piecemeal approach to the implementation and evaluation of interventions regarding such problems is often used. This project will provide comprehensive data on the prevalence of alcohol-related harms over time within definable communities, and community attitudes regarding interventions applied. The evidence from this study can then be used by police and local communities across Australia and internationally to inform the implementation of strategies to reduce violence and disorder around licensed venues and help target police and community resources more effectively and efficiently.

## Conclusion

There is a clear and growing need for more comprehensive evaluations of trends in and attitudes towards interventions targeting harms associated with late night venues and licensed venues more broadly [29]. DANTE is the largest study of its kind to date and will provide important information about the effectiveness of many locally-led interventions as well as alcohol-related harm in the community.

## Competing interests

The authors declare that they have no competing interests.

## Authors' contributions

PM, DP, ND and FdG drafted the background section and details the protocol for venue patron interviews. JT, KG, ASawyer, DG, JW and CL drafted sections detailing venue observations and data analysis, which will be run on all sections. EMcF and DP drafted sections detailing key informant interviews. All authors contributed to proof reading and critical revisions on all sections for the final draft.
